# LSWM: A Long–Short History World Model for Bipedal Locomotion via Reinforcement Learning

**DOI:** 10.3390/biomimetics11010040

**Published:** 2026-01-05

**Authors:** Jie Xue, Zhiyuan Liang, Haiming Mou, Qingdu Li, Jianwei Zhang

**Affiliations:** 1 School of Optical-Electrical and Computer Engineering, University of Shanghai for Science and Technology, Shanghai 200093, China; 221240078@st.usst.edu.cn (J.X.); 231260251@st.usst.edu.cn (Z.L.); 2 Institute of Machine Intelligence, University of Shanghai for Science and Technology, Shanghai 200093, China; 3 Shanghai DroidUp Co., Ltd., Shanghai 200433, China; mhming@droidup.com; 4 Department of Informatics, University of Hamburg, 20146 Hamburg, Germany; jianwei.zhang@uni-amburg.de

**Keywords:** bipedal robot, state reconstruction module, state prediction module, Long–Short World Model

## Abstract

The presence of sensor noise, missing states and inadequate future prediction capabilities imposes significant limitations on the locomotion performance of bipedal robots operating in unstructured terrain. Conventional methods generally depend on long-term history observations to reconstruct single-frame privileged information. However, these methods fail to acknowledge the pivotal function of short-term history in rapid state responses and the significance of future state prediction in anticipating potential risks. The proposed framework is a Long–Short World Model (LSWM), which integrates state reconstruction and future state prediction to enhance the locomotion capabilities of bipedal robots in complex environments. The LSWM framework comprises two modules: a state reconstruction module (SRM) and a future state prediction module (SPM). The state reconstruction module employs long-term history observations to reconstruct privileged information in the current short-term history, thereby effectively improving the system’s robustness to sensor noise and enhancing state observability. The future state prediction module enhances the robot’s adaptability to complex environments and unpredictable scenarios by predicting the robot’s future short-term privileged information. We conducted extensive comparative experiments in simulation as well as in a variety of real-world indoor and outdoor environments. In the indoor stair-climbing task, LSWM achieved a 94% success rate, outperforming the current state-of-the-art baseline methods by at least 34%, thereby demonstrating its substantial performance advantages in complex and dynamic environments.

## 1. Introduction

Reinforcement learning (RL) has demonstrated significant advantages in bipedal robot motion control in recent years, enabling robots to autonomously learn efficient and flexible motion strategies within high-dimensional control spaces. In structured or idealized simulation environments, RL methods have achieved various complex behaviors, including stable walking, rapid running, and dynamic turning [1,2]. However, when these strategies are directly deployed in real-world environments, their performance typically degrades significantly. This phenomenon primarily stems from factors prevalent in real environments, such as sensor noise, unobservable states, and external disturbances, which exacerbate the “sim2real gap” effect [3,4].

In real-world scenarios, sensor data is often subject to uncertainty, measurement accuracy limitations, and environmental interference. Conversely, sensor data in simulation environments is typically idealized and lacks noise [5]. Consequently, policies trained in simulations become overly reliant on the accuracy and stability of sensor data. When deployed in real environments, these policies struggle to effectively handle noise, leading to abrupt changes in policy outputs that compromise walking performance and safety [6]. A common solution involves domain randomization techniques to simulate real-world signal noise distributions in simulations, thereby enhancing strategy robustness [7]. However, determining a reasonable range for noise distribution is challenging. Excessive randomization may hinder training convergence or even prevent obtaining optimal strategies. Furthermore, existing methods often exhibit limitations: for instance, while filtering operations can smooth observation signals, they may compromise the Markov property of reinforcement learning, weakening the policy’s dynamic responsiveness. Conversely, penalizing policy jumps through the reward function is challenging to design, and improper parameter tuning can easily trigger policy oscillations [8].

In RL control frameworks, due to the difficulty of obtaining complete state information in real systems, policy training is typically modeled as a Partially Observable Markov Decision Process (POMDP). The core challenge lies in effectively utilizing history observation sequences to reconstruct a complete state estimate of the environment [9]. To mitigate performance bottlenecks caused by incomplete observations and policy degradation, existing research often incorporates privileged information. Methods such as privileged distillation or history sequence modeling enhance the policy’s robustness against dynamic changes in the actual environment [10]. Specifically, a mainstream approach attempts to infer the privileged state at the current time step from long-term history observations to improve state estimation accuracy. However, these methods typically focus on system identification and dynamic modeling at the current time step, failing to fully leverage the immediate feedback role of short-term history information during rapid state changes. A comparative study by [2] demonstrates that incorporating short-term history observations into policy network inputs significantly improves learning efficiency and simulation-to-reality transfer robustness for various dynamic bipedal skills (e.g., walking, running, jumping) compared to using only current observations. This finding highlights the critical value of short-term history information in complex robotic motion control. However, existing methods generally lack explicit modeling and utilization of future states, hindering the formation of proactive responses to potential risks or dynamic changes. This limits the generalization and adaptability of policies in complex, non-stationary environments. Notably, the literature [10] effectively improved robotic motion performance by introducing predictions of future single-frame states, demonstrating the significant potential of future state information in control decision-making. Based on this, we propose a framework that integrates long-term and short-term history with world modeling. Its core contributions include:

- We propose a LSWM framework. This framework reconstructs noise-free short-term history privileged information from long-term history to address sensor noise and missing state issues. The reconstruction of short-term history privileged information provides explicit feedback for real-time robot control.
- Our SPM can forecast short-term privileged information, enabling robots to make more comprehensive decisions by integrating long-term and short-term history data with future insights.
- We conduct extensive scenario testing both indoors and outdoors.

## 2. Related Work

### 2.1. Legged Robot Locomotion Learning

In RL research for legged robots, learning action mappings directly from high-dimensional observations has become a key paradigm for achieving dynamically robust motion. Such methods automatically discover stable gaits adapted to complex dynamics and multi-contact conditions through interactions in extensive simulated environments [11]. For instance, [12] successfully guided agents to emerge complex locomotive behaviors—including running, jumping, and turning—by introducing periodic reward mechanisms. This demonstrates the critical role of carefully designed tasks and reward functions in facilitating the spontaneous formation of natural gaits. This approach has profoundly influenced subsequent end-to-end motion control research.

Within this framework, effectively utilizing the robot’s intrinsic perception information has become a key research focus. Related methods can be broadly categorized into two types: one is the classical “teacher-student distillation” framework, first proposed by Lee et al. [13]. This method employs a two-stage training process: first, training a teacher policy that has access to privileged information; subsequently, the student policy mimics the teacher’s actions through behavior cloning, relying solely on intrinsic perception. This mechanism achieves performance comparable to the teacher policy with minimal sensory input, leading to its adoption and refinement in subsequent studies [14,15,16]. Another class of research focuses on simplifying the training process, proposing alternatives requiring only a single-stage training phase [17]. For instance, DreamWaQ employs an asymmetric actor-critic architecture to avoid explicit reliance on privileged information, enabling efficient policy learning based solely on self-perception [10].

Meanwhile, the integration of imitation learning and reinforcement learning has also driven significant improvements in the quality of motion control. The literature [18] generated highly anthropomorphic gaits by integrating AMP with periodic rewards; DeepMimic systematically leveraged reference motions to guide rewards, enabling agents to learn high-quality dynamic actions with recovery capabilities, such as walking and rolling [19]. These methods have been widely adopted in robotic gait imitation and skill transfer [20], while BeyondMimic further validated its robust motion generation and adaptation capabilities on real robotic platforms [21].

### 2.2. State Reconstruction and State Prediction

One mainstream research approach focuses on reconstructing the privileged state at the current moment from long-term history observation sequences to enhance state estimation accuracy. For instance, references [22,23] propose jointly encoding the robot’s intrinsic state and environmental information as latent variables, leveraging temporal modeling to recover the system state. To further improve reconstruction efficiency, ref. [24] explores an architecture for concurrent training of policy networks and state estimators; ref. [25] proposes a hierarchical reconstruction strategy that explicitly estimates root node velocity while encoding remaining states as latent variables. However, most of these approaches focus on system identification and dynamic modeling at a single timescale, failing to effectively utilize short-term history privileged information to address sudden state changes. Furthermore, existing methods generally lack the ability to model future state evolution trends, making it difficult to provide forward-looking decision support for control systems.

In contrast, state prediction methods aim to construct dynamic models of the environment to forecast future states. The concept of world models was introduced in [26], focusing on constructing internal representations of the environment to support temporal decision-making. Subsequent research includes the Dreamer series of algorithms, which fuse long-term and short-term state estimates through hierarchical prediction mechanisms [10]; Daydreamer emphasizes instantaneous prediction during online learning to reduce trial-and-error costs in practical interactions [27]. The denoising world model (DWL) reconstructs and predicts single-frame privileged states using long-term history observations during denoising [28]. Nevertheless, existing prediction methods primarily focus on estimating states at a single future time step, failing to fully leverage the holistic value of short-term history privileged states in sequential decision-making.

## 3. Method

**Figure 1:**
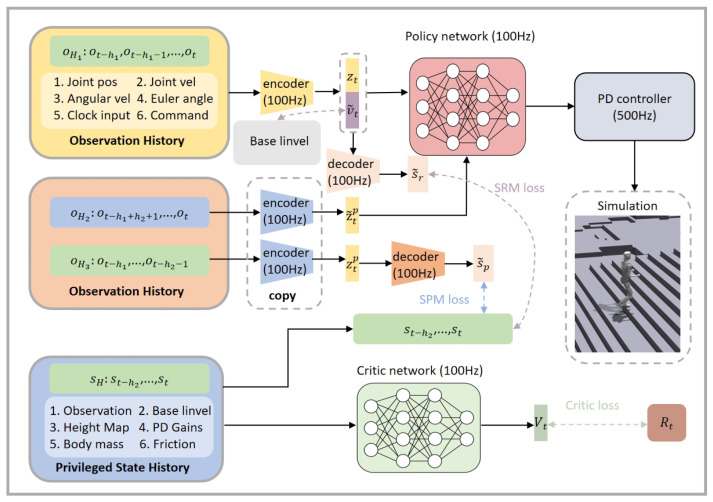
LSWM framework. LSWM simultaneously models short-term history and future states to improve the quality of state reconstruction and enhance the policy’s forward-looking adaptability. The asymmetric actor-critic structure enables the SRM, SPM, and control policy to be jointly optimized within a single training phase.

Unlike existing methods that rely solely on long-term history information for single-frame state reconstruction, LSWM simultaneously models short-term history states and future states to fully leverage the dynamic characteristics of recent history and the trend information of future states. Through the following two core submodules, LSWM enhances the quality of current state observation while maintaining policy responsiveness, and strengthens its forward-looking adaptive capabilities. We adopt an asymmetric actor-critic framework, as illustrated in Figure 1. Consequently, we require only a single training phase to optimize both modules and the policy network.

### 3.1. Reinforcement Learning Task for Bipedal Locomotion

In this study, we model the task of bipedal locomotion over complex terrains as a POMDP with discrete time steps t∈N, defined as

(1) M={S,O,A,T,Z,R,γ},

where S, O, and A denote the state, observation, and action spaces, respectively.

The state transition probability $T(s,a,s^{\prime })$ represents the likelihood of reaching a new state $s^{\prime }$ after executing action *a* in state *s*; the observation probability $Z(s^{\prime },a,o)$ represents the probability of observing *o* after executing action *a* and transitioning to state $s^{\prime }$; the reward function $R(s,a,s^{\prime })$ denotes the immediate reward obtained after taking action *a* in state *s* and arriving at state $s^{\prime }$; and the discount factor γ∈[0,1] balances the relative importance of immediate and future rewards.

The objective of RL is to find a policy $\pi (a_{t}|o_{t})$ that maximizes the expected discounted cumulative reward:

(2) $$J\left(\pi \right)=E_{\pi }\left[\sum_{t=0}^{\infty }\gamma^{t}r_{t}\right],$$

where $r_{t}=R\left(s_{t},a_{t},s_{t+1}\right)$ is the immediate reward at time step *t*.

### 3.2. State Reconstruction Module

State Reconstruction Module: The SRM is a component within a larger system. Given the partial observability of the environment, robots cannot access complete state information during decision-making. The partial observations relied upon by the system often reflect only local environmental features, failing to capture overall dynamic changes. This limits the system’s ability to perform precise perception and effective decision-making in complex environments. Therefore, this module’s responsibility is to reconstruct short-term privileged history information from noisy long-term history observations. Noise in long-term history observations is illustrated in Table 1. Such privileged information contains no redundant content and includes state information unobtainable in the real world. This approach mitigates the impact of noise on the policy and enhances the observability of policy inputs. Research indicates that short-term history data can coordinate with the adaptability generated by long-term history data, thereby promoting improvements in real-time control performance. Furthermore, explicit estimation of linear velocity has been demonstrated to enhance the tracking accuracy of robotic velocity commands. SRM consists of an encoder-decoder structure, as shown in Figure 1. The encoder $E_{SRM}\left({\tilde{v}}_{t},z_{t}|o_{H_{1}}\right)$ takes long-term history observations $o_{H_{1}}$ as input and outputs the estimated linear velocity ${\tilde{v}}_{t}$ and implicit features $z_{t}$. SRM estimates the linear velocity $v_{t}$, effectively enhancing the robustness of the strategy, with its key advantage being improved tracking accuracy of the velocity command. The decoder $D_{SRM}\left({\tilde{s}}_{r}|z_{t},v_{t}\right)$ takes $v_{t}$ and $z_{t}$ as input and outputs the reconstructed short-term history privileged information ${\tilde{s}}_{r}$. $o_{t}$ is defined as

(3) $$o_{t}={\left[phase\;cmd\;q_{t}\;{\dot{q}}_{t}\;\Phi \;\omega \right]}^{T},$$

where, phase, cmd, $q_{t}$, ${\dot{q}}_{t}$, Φ, and ω are the phase information, velocity commands, joint positions, joint velocities, body Euler angles, and body angular velocities, respectively.

$s_{t}$ is defined as

(4) $$s_{t}={\left[\begin{array}{ccc} o_{t} & v_{t} & h_{t} \end{array}\right]}^{T},$$

where, $v_{t}$ is linear velocity. $h_{t}$ represents privileged information such as elevation maps and friction coefficients.

$z_{t}$ represents the feature extraction from the long-term history $o_{H_{1}}$. The loss function of the SRM is defined as

(5) $$L_{\mathrm{SRM}}=MSE\left({\tilde{v}}_{t},v_{t}\right)+MSE\left({\tilde{s}}_{r},s_{H}\right),$$

where, ${\tilde{v}}_{t}$ is the reconstructed linear velocity. ${\tilde{s}}_{r}$ is the reconstructed short-term history privileged information.

### 3.3. State Prediction Module

In highly dynamic tasks such as bipedal walking, robots need to achieve stable and flexible movement in rapidly changing environments, which imposes high demands on the foresight and decision-making capabilities of their strategies. To address this challenge, the SPM models the potential state transitions of the robot during action execution, enabling the prediction of the possible physical states and their evolution under different action choices. The key advantage of SPM lies in its foresight modeling capability, which allows the robot to proactively assess the potential consequences of different behaviors before making decisions, thus enabling value-driven decision-making rather than relying solely on immediate rewards for policy optimization. The core idea of SPM is to use history observation data to reconstruct short-term future states. The goal is not to precisely estimate every detail of future states but to predict future trends, helping the robot make adaptive adjustments to environmental changes during action execution. Through this predictive ability, the robot can plan for upcoming state changes in advance, thereby improving overall motion performance and task execution stability. Specifically, the encoder $E_{SPM}\left(z_{t}^{p}|o_{H_{3}}\right)$ takes a sequence of history observations $o_{H_{3}}$ as input and maps it to compact latent features $z_{t}^{p}$. These latent features not only reduce the dimensionality of the original history information but also preserve dynamic information closely related to future state evolution. Subsequently, the decoder $D_{SPM}\left({\tilde{s}}_{p}|z_{t}^{p}\right)$ transforms the latent features $z_{t}^{p}$ into reconstructed short-term privileged history information ${\tilde{s}}_{p}$, thereby simulating the robot’s state evolution over the next several time steps. The significance of this reconstruction process lies in its ability to provide predictions about future states, thereby enabling the policy network to consider potential state evolutions during decision-making. For instance, the robot can anticipate risk events such as joint hyperextension, foot tip slippage, or height instability during specific gaits, enabling preventive adjustments in action selection. By integrating the predicted latent features with current short-term history information, the robot obtains a comprehensive representation encompassing both recent dynamic trends and potential future state information, thereby achieving safer, more informed, and robust decision-making. Furthermore, SPM can be jointly trained with policy networks within reinforcement learning frameworks. During training, by minimizing the error between predicted features and actual short-term privileged information, robots learn compact yet information-rich dynamic latent space representations. This not only enhances state estimation accuracy but also significantly improves policy adaptability in complex terrains, nonlinear dynamics, and under external disturbances.

The loss function of the SPM is defined as

(6) $$L_{\mathrm{SPM}}=MSE\left({\tilde{s}}_{p},s_{H}\right),$$

where, ${\tilde{s}}_{p}$ represents the predicted short-term history privileged information.

Policy Learning: We adopt an asymmetric actor-critic framework. Specifically, the critic can directly access privileged information ${\tilde{s}}_{H}$ from the simulation environment, while the policy network takes as input the implicit information $z_{t}$, ${\tilde{z}}_{t}^{p}$, and ${\tilde{v}}_{t}$ output by the SRM and SPM modules, and learns the corresponding action policy $\pi (a_{t}|z_{t},z_{t}^{p},{\tilde{v}}_{t})$. ${\tilde{z}}_{t}^{p}$ is a copy of the encoder’s $E_{SPM}$ parameters. The generation process is $E_{SPM}\left({\tilde{z}}_{t}^{p}|o_{H_{2}}\right)$. During training, Proximal Policy Optimization (PPO) is used, maximizing the expected cumulative return via multi-step stochastic gradient descent. The optimization objective of the policy can be expressed as the following loss function:

(7) $$\begin{array}{cc} L_{\pi }= & \min(\frac{\pi_{\theta }\left(a|z_{t},z_{t}^{p},{\tilde{v}}_{t}\right)}{\pi_{\theta_{b}}\left(a|z_{t},z_{t}^{p},{\tilde{v}}_{t}\right)}A^{\pi_{\theta_{b}}},\\ \; & \;\;\;\mathrm{clip}\left(\frac{\pi_{\theta }\left(a|z_{t},z_{t}^{p},{\tilde{v}}_{t}\right)}{\pi_{\theta_{b}}\left(a|z_{t},z_{t}^{p},{\tilde{v}}_{t}\right)},\;1-\epsilon ,\;1+\epsilon \right)A^{\pi_{\theta_{b}}}), \end{array}$$

where, *a* represents the action generated by the policy. π denotes the current optimized policy, while $\pi_{b}$ is the policy used in the previous iteration. ϵ is a hyperparameter that controls the range of divergence between the new and old policies, thereby limiting the magnitude of policy updates and preventing overfitting. The advantage function $A^{\pi_{\theta_{b}}}$ represents the advantage output by the policy network during the previous training round, whose computation relies on the value function $V_{\phi }\left(s\right)$. When updating the value function, the following optimization objective is followed:

(8) $$L_{v}={\left(V_{\phi }\left(s_{t}\right)-{\hat{R}}_{t}\right)}^{2}.$$

At time *t*, $R_{t}$ denotes the cumulative return obtained by the agent, while $V(s_{t})$ represents the value predicted by the critic for the current state $s_{t}$.

The loss function of LSWM is defined as

(9) $$L_{\mathrm{LSWM}}=L_{\mathrm{SRM}}+L_{\mathrm{SPM}}+L_{\pi }+L_{v},$$

where, $L_{\mathrm{SRM}}$ is the reconstruction loss. $L_{\mathrm{SPM}}$ is the prediction loss. $L_{\pi }$ is the policy loss. $L_{v}$ is the value loss.

### 3.4. Action Space and Reward Design

In this paper, the bipedal robot operates in a continuous action space, where the action vector $a_{t}$ consists of incremental target positions for each joint, totaling 10 dimensions (corresponding to 10 controllable joints). The reward function is designed to guide the robot toward stable and efficient walking while avoiding dangerous or unnatural movements. The foot swing and stance penalties originate from Reference [12]. The overall reward is shown in Table 2.

## 4. Environment Setup

**Figure 2:**
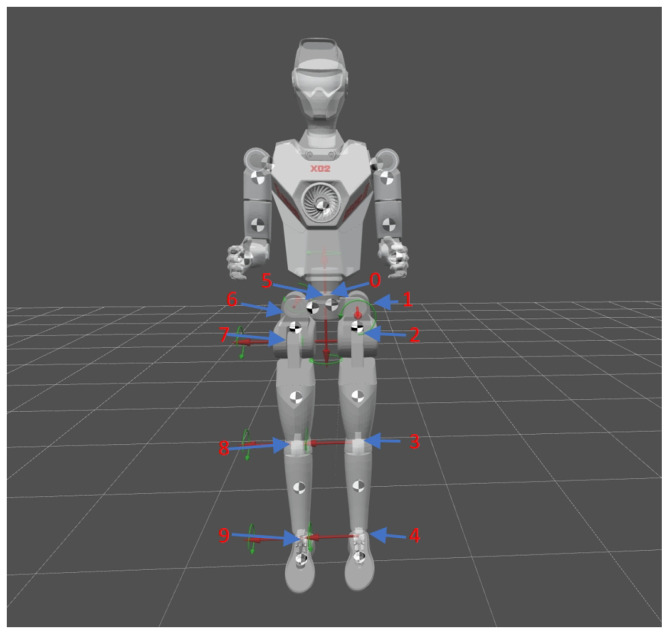
X02 Robot Structure.

The robot platform used in this experiment is the X02 standard version, developed by Shanghai Droid. The X02 robot stands at a height of 1.7 m, weighs 32 kg, and has 20 degrees of freedom. In this simulation training, we controlled only the 10 degrees of freedom of the lower body. The degrees of freedom of the lower body and the corresponding centroid positions of each body part are shown in Figure 2. The range of motion for each joint is shown in Table 3.

**Figure 3:**
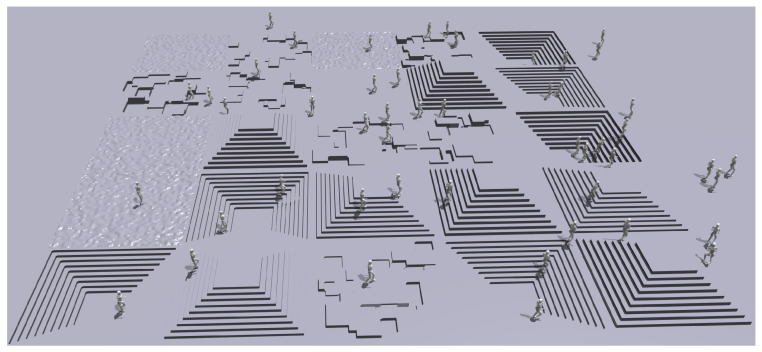
Representative terrain types designed in the simulation environment. Five representative terrains, including slopes, flat surfaces, pyramid-shaped stairs, recessed stairs, and discrete obstacles, are constructed to evaluate the robot’s adaptability and generalization across diverse terrains.

To enable robust motion control across diverse terrains, we construct a large-scale parallel reinforcement learning training environment based on the Isaac Gym simulation platform. In this system, 4096 agents operate concurrently, fully leveraging the parallel computing capabilities of GPUs to significantly accelerate policy convergence. To improve the strategy’s adaptability and generalization in real-world scenarios, we design five representative terrain types within the simulation: slopes, flat surfaces, pyramid-shaped stairs, recessed stairs, and discrete obstacles, as illustrated in Figure 3. During the initial training phase, all agents start on the simplest terrain. Once an agent can reliably traverse its current terrain, its training difficulty is adaptively increased to more complex scenarios, implementing a curriculum learning mechanism that progresses from easy to hard. This approach effectively mitigates convergence instability in the early training stages and enhances the policy’s generalization performance across diverse terrains.

For time parameter settings, each training episode lasts a maximum of 24 s, with a control loop frequency of 100 Hz, corresponding to 100 state updates per second. All joints are controlled using PD controllers, with proportional and derivative gains set to 100 and 2, respectively. To reduce impact forces at the feet, the ankle joint gains are slightly lower, set to 20 and 1. We adopt PPO as the core optimization algorithm, which efficiently explores the policy space while ensuring stable training. This enables the learning of motion control strategies that exhibit strong transferability and dynamic stability across complex and variable terrains. The PPO algorithm and its key hyperparameters are summarized in Table 4.

## 5. Results

To evaluate the effectiveness of the LSWM method, we conducted extensive walking tests both indoors and outdoors and performed comparative experiments on the following methods:

- LSWM: Our method.
- DWL: Reconstruction performed only on single-frame privileged information.
- SRM: Reconstruction performed only on multi-frame privileged information.
- DreamWaq: Observation that predicts only the next step into the future.
- SRPM: Reconstructed multi-frame privilege information and predicted the state for the next step.

**Figure 4:**
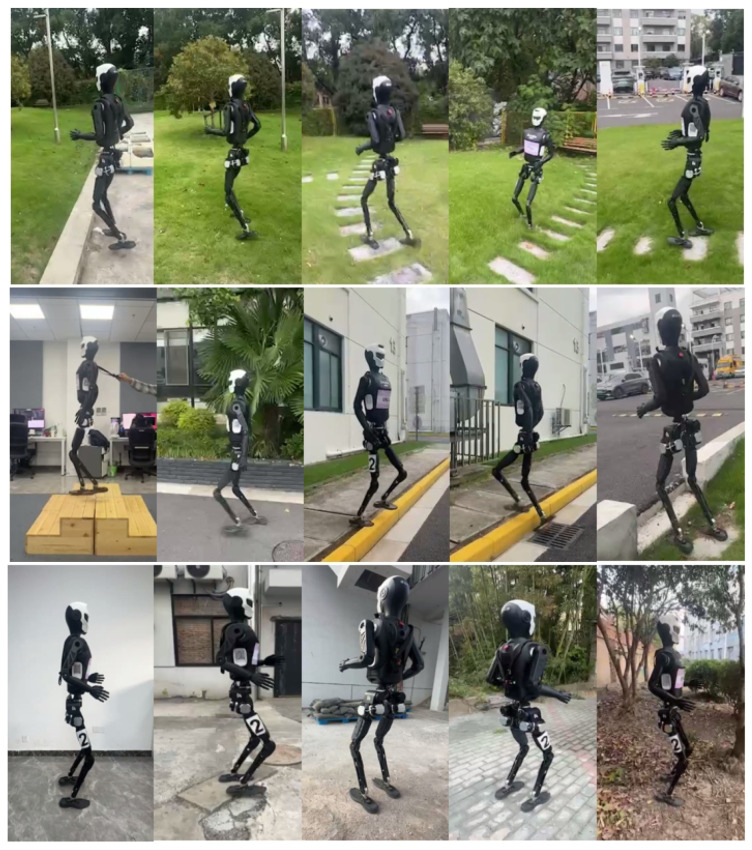
Indoor and outdoor testing scenarios. The terrain includes asphalt surfaces, 20 cm discrete obstacles, grassy areas, two-step 16 cm stairs, slopes, muddy ground, sandy areas, smooth surfaces, and uneven stone slabs.

**Figure 5:**
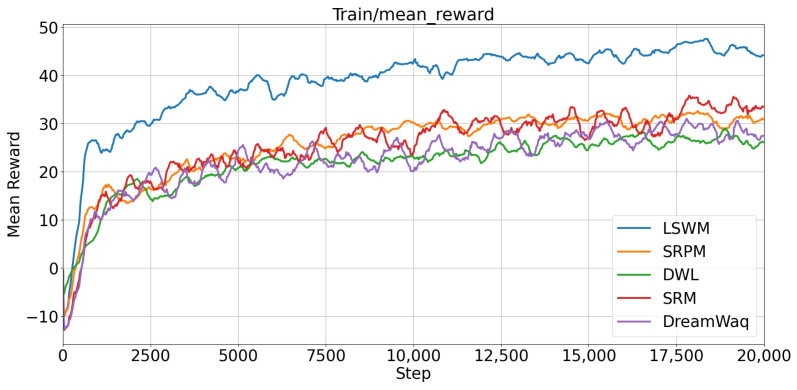
Average reward trends of different algorithms during training.

The indoor and outdoor test scenarios are shown in Figure 4. To further analyze the performance evolution of different methods during training, the trends of average rewards with respect to training steps are illustrated in Figure 5. All five algorithms were trained for 20,000 iterations using the same reward function. As shown in the figure, LSWM exhibits a significantly faster reward growth rate than all comparison methods. During the early training phase (approximately 0–500 iterations), LSWM demonstrates a notably steeper reward-increase slope and surpasses the final convergence levels of other methods within around 1000 iterations. It is worth noting that we applied exponential moving average smoothing to the average reward and the average reward for linear velocity, with a smoothing parameter set to 0.25.

**Figure 6:**
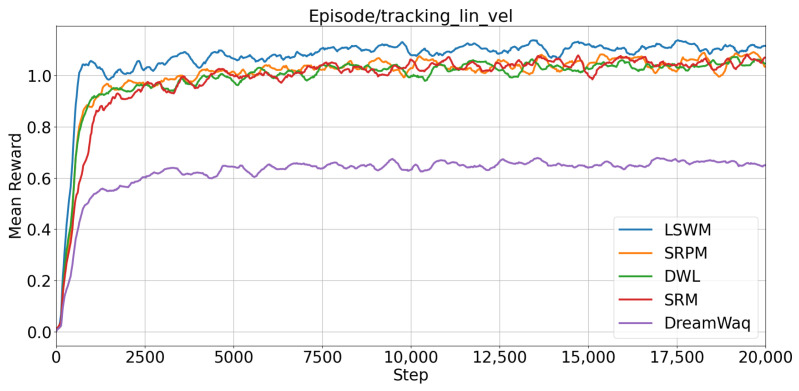
Comparison of linear velocity tracking rewards among different algorithms.

The average reward for linear velocity tracking is presented in Figure 6. The results indicate that LSWM achieves the fastest improvement in tracking reward, clearly outperforming the other methods. The reward growth trends of SRPM and SRM are similar, with SRPM converging slightly faster than SRM, and both outperforming DreamWaq and DWL. These observations suggest that the incorporation of short-term history privileged information and future-state prediction in the SPM module substantially accelerates policy convergence. Specifically, the SPM encodes short-term history privileged states and predicts future dynamics, enabling the policy network to anticipate the potential impacts of its actions on both the robot and the environment. Consequently, the policy obtains stronger reward signals during the early stages of training, thereby speeding up the learning of linear velocity tracking. In contrast, DreamWaq does not utilize history information, and DWL only exploits single-frame privileged information. As a result, both methods exhibit slower reward improvement and weaker adaptability to complex terrains compared with SRPM, SRM, and LSWM, which benefit from the use of short-term history privileged information.

**Figure 7:**
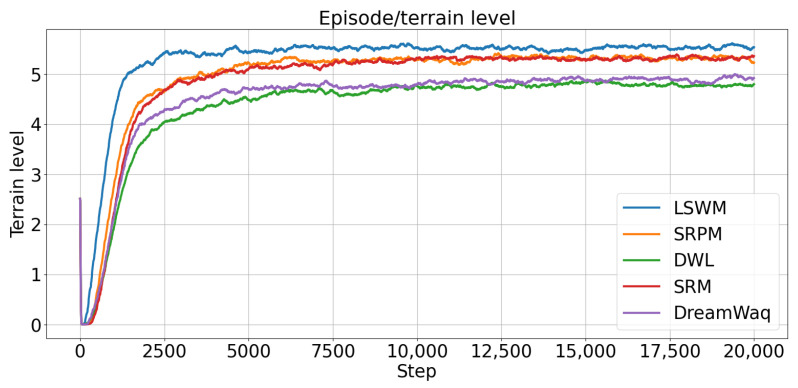
Evolution of the average terrain difficulty level during training.

Figure 7 illustrates the variation of the average terrain difficulty level during training. LSWM rapidly reaches higher terrain difficulty levels in the early training phase, demonstrating its superior capability to adapt to complex environments. SRPM and SRM exhibit moderate improvement rates, outperforming DreamWaq and DWL. This indicates that faster reconstruction of short-term history states provides a more rapid response to terrain variations under blind vision conditions. However, SRPM and SRM still lag slightly behind LSWM. This trend highlights the critical importance of short-term history privileged information and future-state prediction: the SPM module enables the policy to anticipate the potential outcomes of its actions in complex environments, allowing the agent to traverse challenging terrains more safely and efficiently.

**Figure 8:**
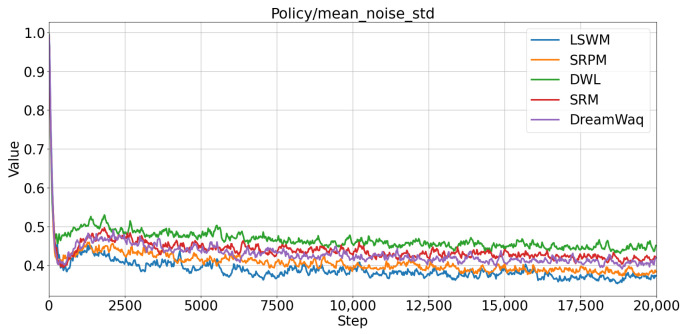
Training stability: mean action noise standard deviation.

The mean noise standard deviation curves of the five algorithms are shown in Figure 8. Although all five methods eventually converge to stable levels, LSWM reaches the lowest and most stable noise level the fastest (approximately 0.5 after 5k steps), demonstrating superior stability.

**Figure 9:**
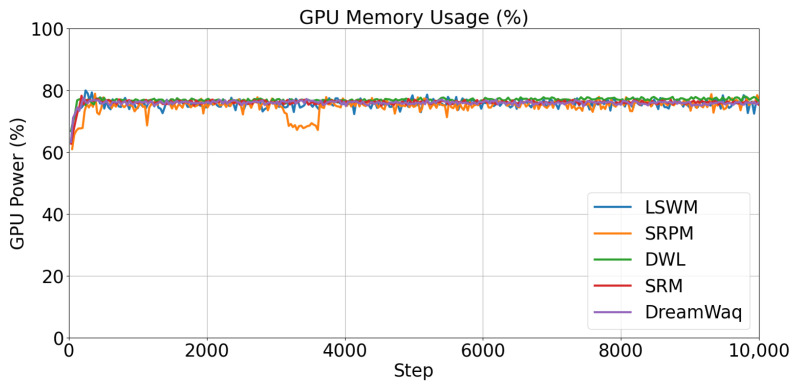
Percentage of GPU Memory Usage.

In terms of training time, DWL completed 20,000 training epochs in approximately 3 h and 33 min, SRM took about 3 h and 46 min, DreamWaq took around 3 h and 40 min, SRPM took approximately 4 h, and LSWM took about 4 h and 20 min. The GPU utilization of the five algorithms is shown in Figure 9. The figure indicates that the GPU usage patterns are largely consistent across all five algorithms. Although LSWM exhibits relatively longer training times, our primary focus remains on the robot’s actual walking performance, strategy stability, and generalization capability in complex terrain. Next, we conduct comparative testing of our methods in real-world scenarios.

**Figure 10:**
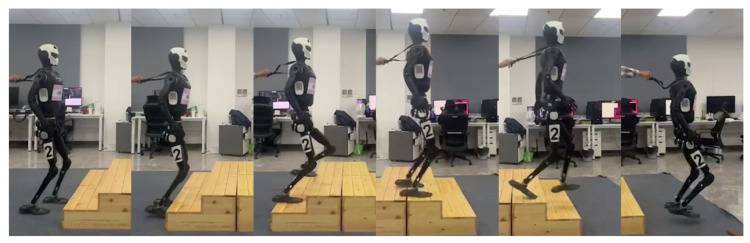
Indoor stair-walking test environment used for evaluating stability and generalization. The scenario includes two ascending and two descending steps (16 cm height each) with a flat transition platform.

In indoor experiments, we construct a complex walking test environment with ascending and descending staircases to evaluate the stability and generalization capabilities of different algorithms on terrain with height variations. Specifically, the scenario consists of two ascending stair steps (16 cm per step) and two descending stair steps (16 cm per step), separated by a short horizontal transition platform to simulate real-world stair connections, as shown in Figure 10. Within this environment, we systematically test five distinct control methods. Each algorithm is independently evaluated 10 times under identical initial conditions and target velocity commands to ensure comparability and statistical significance. A failure is recorded if the robot falls, experiences significant slipping causing postural instability, or fails to complete the stair-climbing task. Table 5 summarizes the statistics of falls, slips, stair-climbing failures, and overall success rates across the 50 trials for each method. The results indicate that LSWM successfully completes all trials, demonstrating the highest stability and robustness. SRPM and SRM also achieve high success rates, with SRPM slightly outperforming SRM. In contrast, DreamWaq and DWL frequently fall or lack sufficient climbing momentum during ascent and descent, resulting in considerably lower success rates. The primary reason for this difference is that LSWM and SRPM both incorporate short-term history privileged information and explicitly model dynamic changes of future states through the SPM module. This allows the policy to anticipate the dynamic impacts of terrain undulations in advance, maintaining stable gait transitions on discontinuous terrain such as stair edges. Compared to SRPM, LSWM further integrates joint optimization of short-term history encoding and future state prediction, establishing stronger temporal correlations within the state representation space. SRM, relying solely on short-term history encoding without explicit future state prediction, shows slightly inferior stability during stair transitions. DreamWaq, which does not use history information, and DWL, which only leverages single-frame privileged information, cannot promptly adjust foot trajectories and trunk posture when encountering abrupt terrain changes, leading to insufficient ascent momentum and increased instability during descents.

**Figure 11:**
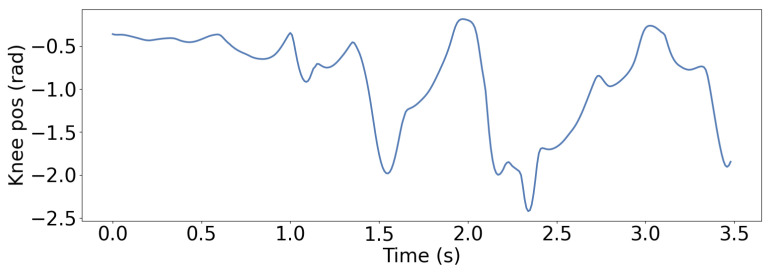
Knee joint trajectory variations of LSWM during stair-climbing.

**Figure 12:**
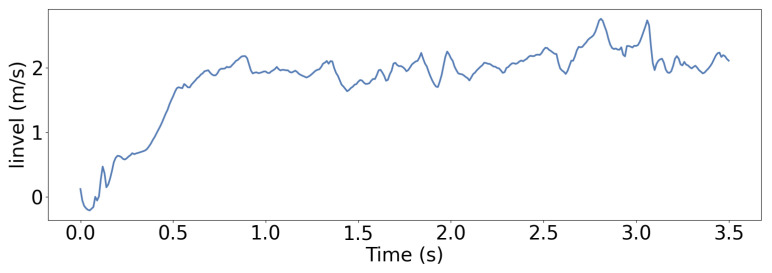
Linear velocity tracking performance of LSWM during stair-climbing.

In the stair-climbing experiment, the gait behavior and knee joint trajectory changes of LSWM are shown in Figure 11. The figure reveals that the robot maintains a low foot lift height during the flat ground phase, with the foot elevation significantly below the stair step height. Upon first contact with the stair edge, the robot’s strategy rapidly adjusts the foot trajectory, markedly increasing the lift height to successfully clear the step edge without collision. Subsequently, the robot maintains a large foot lift amplitude during the continuous ascent phase to ensure the foot tip can smoothly traverse each step. Upon entering the intermediate transition platform, the robot proactively reduces the foot lift height again, reverting to the flat-ground walking mode. During the subsequent descent phase, the knee joint angle gradually decreases, achieving a smooth transition through early gait adjustment. Throughout stair climbing, the robot continuously adjusted stride length and frequency based on given linear and angular velocity commands, achieving precise target velocity tracking. Even with significant step height variations, velocity error remained within a narrow range, as shown in Figure 12. This demonstrates the state reconstruction and prediction capabilities of the LSWM method.

In the staircase tests, we further analyzed the contributions of SRM and SPM to the overall performance. SRM reconstructs the current state from multi-frame history privileged information, enhancing action smoothness and gait stability while mitigating the effects of noise and short-term disturbances. SPM predicts future states, allowing the policy to proactively adjust actions in response to terrain variations, thereby improving its anticipatory adaptability. When combined in LSWM, the policy benefits from both stable state estimation and foresight into future dynamics, achieving comprehensive improvements in action smoothness, stability, and energy efficiency. Experimental results demonstrate that SRM and SPM play complementary roles within LSWM: SRM ensures reliability of the current state, while SPM provides foresight, and their synergy significantly enhances the robot’s stability and generalization capability in complex terrains.

**Figure 13:**
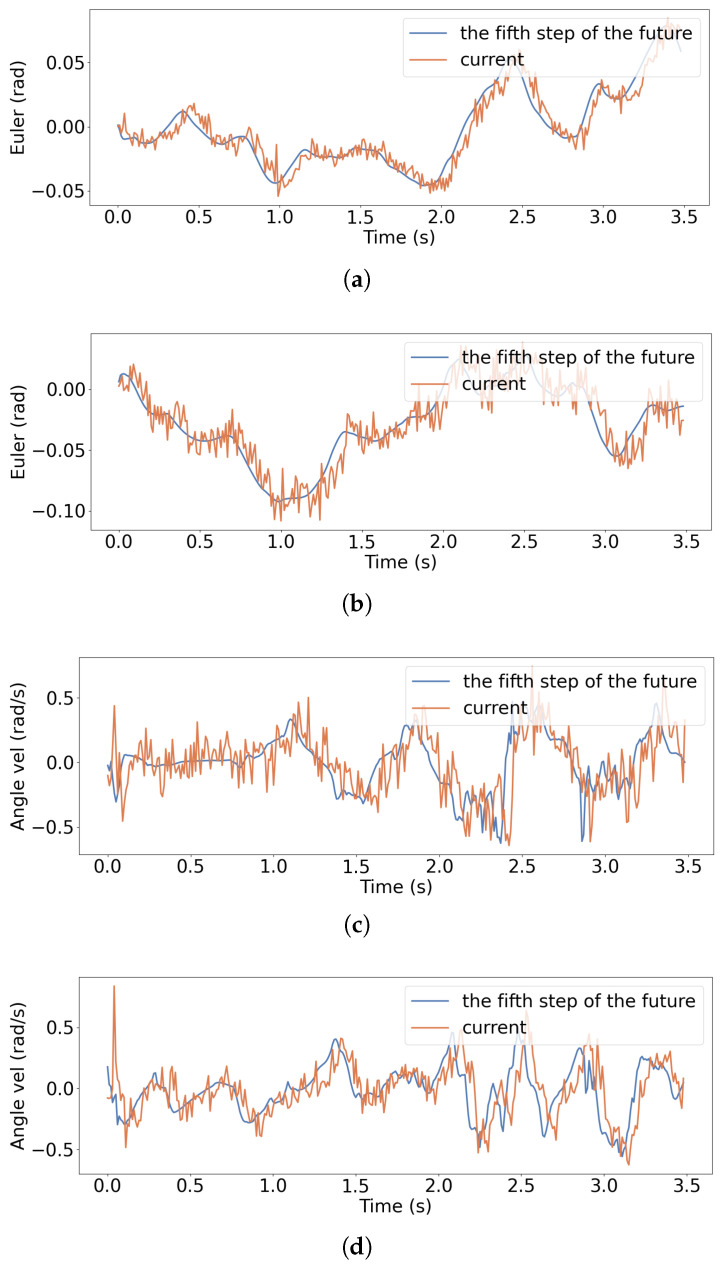
Comparison between actual and predicted roll/pitch Euler angles and angular velocities during stair climbing: (**a**) Roll angle; (**b**) Pitch angle; (**c**) Roll angular velocity; (**d**) Pitch angular velocity.

We further evaluate the variations of roll and pitch Euler angles and their angular velocities during the stair-climbing process, together with the corresponding predictions generated by the SPM module, as illustrated in Figure 13. By comparing the trajectories of the actual and predicted Euler angles and angular velocities, the SPM’s capability in dynamic attitude prediction can be more directly assessed. The actual Euler angles and angular velocities were obtained from the onboard IMU. Although there is noise in the data, it still reflects the changes in the robot’s current state. The MSE between the euler angles of roll and pitch and their angular velocities and the predicted values are 0.0001, 0.0002, 0.0415, and 0.0359, respectively. The results show that, although the predicted Euler angle and angular velocity curves do not match the real values precisely, their overall trends are highly consistent with the actual changes. SPM is able to effectively capture the robot’s future dynamic changes in body pitch and roll during stair climbing, demonstrating its ability to predict posture evolution trends in complex terrains.

**Figure 14:**
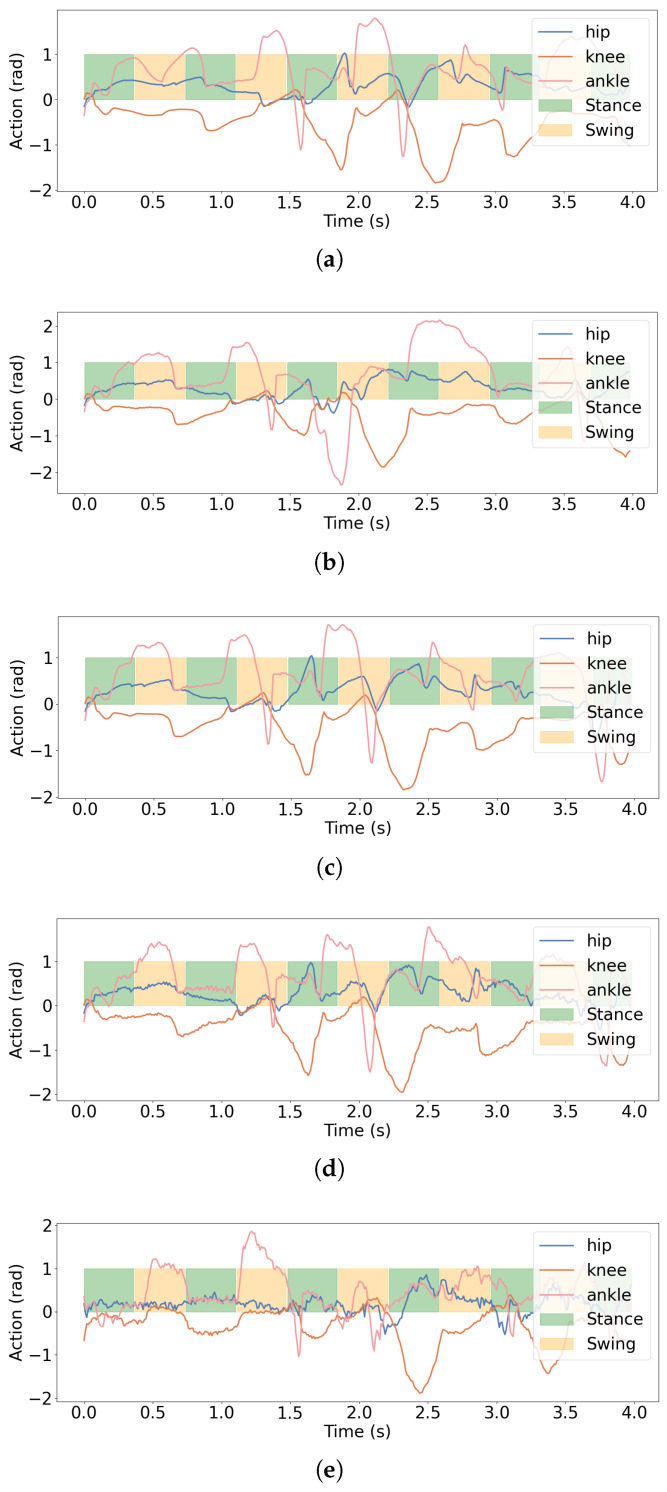
Action outputs of the hip, knee, and ankle joints over 400 walking steps for five algorithms: (**a**) LSWM, (**b**) SRPM, (**c**) SRM, (**d**) DWL, (**e**) DreamWaq. LSWM exhibits the smoothest motions across all joints, while SRPM and SRM show minor oscillations, and DWL and DreamWaq display more significant fluctuations.

We further record the policy network action outputs for the hip, knee, and ankle joints during 4 s across five algorithms, as illustrated in Figure 14. The results indicate that the LSWM algorithm exhibits highly smooth motion across all three joints with no significant oscillations. The SRPM and SRM algorithms, which incorporate state reconstruction modules, show only minor oscillations at isolated moments. Comparing SRM and DWL, which differ only in whether multi-frame state reconstruction is performed, SRM’s action smoothness is significantly superior to DWL’s. Meanwhile, DWL’s action smoothness outperforms DreamWaq, which does not perform state reconstruction. Although both LSWM and SRPM incorporate future state prediction mechanisms based on the SRM module, SRPM predicts only single-frame future states, resulting in slightly lower smoothness than LSWM. These results indicate that the SRM module significantly enhances policy motion smoothness through multi-frame state reconstruction, while the SPM module provides a marginal improvement in smoothness.

Based on the above analysis of joint action smoothness, we further calculated the energy consumption metric Cost of Transport (COT) for the five algorithms during 4 s of stable walking [29], as shown in Table 6. It can be observed that algorithms with smoother action outputs generally exhibit lower energy consumption, which is consistent with the generation of more stable joint torques during control. Among them, LSWM has the smoothest hip, knee, and ankle joint action trajectories, and therefore achieves the lowest COT value, demonstrating the highest energy efficiency. Overall, SRM is the key factor influencing energy consumption, significantly reducing COT during walking, while SPM provides further but modest improvements. By combining both, LSWM achieves the best performance in terms of both action smoothness and energy efficiency.

We conducted extended walking tests on the robot across multiple outdoor scenarios, as shown in Figure 4. Test environments included asphalt pavement, two 16 cm high steps, 20 cm discrete obstacles, grassy terrain, slopes, muddy ground, sandy surfaces, smooth flooring, and uneven stone slabs—effectively covering typical terrain types found in real-world settings. Experimental results demonstrate that the LSWM method achieves stable walking across these diverse scenarios while maintaining strong adaptability and robustness during abrupt terrain transitions. Notably, grass, asphalt, muddy ground, sand, and stone slabs were not included in simulations, yet the robot still performed stable motion in these environments. This fully validates the LSWM method’s superior generalization capability in real-world settings.

The above experimental results indicate that LSWM demonstrates superior performance across a variety of complex indoor and outdoor terrains. Compared with DWL, SRM, DreamWaq, and SRPM, LSWM achieves faster policy convergence, higher reward levels, and greater gait stability, with a 94% success rate in abrupt terrain changes such as staircases. By integrating short-term history privileged information with future-state prediction, the SPM module enables the policy to anticipate the effects of actions in advance, enhancing early-stage reward signals and improving both linear velocity tracking and gait smoothness. Joint action outputs and posture predictions further show that LSWM effectively adapts to terrain variations while maintaining stable locomotion. Extended outdoor tests confirm its generalization capability to previously unseen terrains. Overall, the LSWM framework, which combines multi-frame state reconstruction and future-state prediction, significantly improves learning efficiency, terrain adaptability, and policy robustness, demonstrating clear advantages over existing methods.

## 6. Conclusions

This paper proposes an RL framework based on the LSWM to address the challenges faced by bipedal robots in unstructured and complex terrains, such as sensor noise, missing states, and insufficient future prediction capabilities. By integrating an SRM and an SPM, the framework achieves efficient reconstruction of observational information and reasonable short-term future state prediction, thereby significantly improving the stability and generalization capability of the learned policy. For experimental validation, the proposed LSWM framework was systematically evaluated in both simulation and real-world scenarios. The results demonstrate that the method enables stable locomotion across various complex terrains—including stairs, discrete obstacles, slopes, and grass—and achieves superior performance in terms of motion smoothness, state estimation accuracy, and environmental adaptability compared with existing approaches. Particularly, in real-world outdoor tests involving unseen terrains, LSWM exhibited outstanding generalization and dynamic adaptability, confirming the practicality and effectiveness of the proposed method. In future research, we plan to leverage the predictive capabilities of the SPM module to implement system-level safety warnings, and further explore the integration of LSWM with external perception modules to achieve end-to-end intelligent locomotion from environmental perception to decision-making control. In addition, our current method may occasionally step on the edges of stairs during descent, and we also plan to address this issue by incorporating external perception modules, thereby further enhancing the robot’s safety and stability on unstructured terrains. In the future, we will further explore the theoretical foundations of the LSWM framework through theoretical derivation, thereby revealing the contributions of the dual-temporal world model to system stability.

## Figures and Tables

**Table 1:** Domain Randomization Setting.

Parameter	Unit	Range	Operator
Joint pos noise	rad	[−0.05 , 0.05]	additive
Joint velocity noise	rad/s	[−2, 2]	additive
Euler noise	rad	[−0.05, 0.05]	additive
Angular velocity noise	rad/s	[−0.5, 0.5]	additive
COM	cm	[−0.075, 0.075]	-
Payload mass	kg	[−5, 5]	additive
Friction	-	[0.1, 2]	-
Kp, Kd noise	-	[0.85, 1.15]	additive

**Table 2:** The reward function.

Reward Term	Equation	Weight
Lin. velocity tracking	$\exp\left(-4∥v_{xy}^{\mathrm{cmd}}-v_{xy}{∥}_{2}^{2}\right)$	1.5
Ang. velocity tracking	$\exp\left(-4{\left(\omega_{z}^{\mathrm{cmd}}-\omega_{z}\right)}^{2}\right)$	1
Lin. velocity *z*	$v_{z}^{2}$	−0.5
Ang. velocity (xy)	$∥\omega_{xy}{∥}_{2}^{2}$	−0.3
Orientation (xy)	$∥g_{xy}{∥}_{2}$	−5
Base height	${\left(h_{\mathrm{des}}-h\right)}^{2}$	−20
Joint Torque	${∥\tau ∥}_{2}^{2}$	$-1\times 10^{-5}$
Joint acceleration	${\ddot{q}}^{2}$	$-2.5\times 10^{-7}$
Joint power	$|\tau \dot{q}|$	$-2\times 10^{-5}$
Action rate	$∥a_{t}-a_{t-1}{∥}_{2}^{2}$	−0.01
Feet distance	$\max\left(0,\;0.2-{∥p_{y}^{l}-p_{y}^{r}∥}_{2}\right)$	−10
Hip joint deviation	$\sum_{i\in \mathrm{hip}}{\left|\theta_{i}-\theta_{i}^{\mathrm{default}}\right|}^{2}$	−5
Feet stance	$r_{\mathrm{contact}}$	−2
Feet swing	$r_{\mathrm{swing}}$	−2
Feet contact force	$\mathrm{CLIP}(F_{L,R}-180,0)$	−0.01
Joint limit	$n_{\mathrm{limitation}}$	−5
Collision	$n_{\mathrm{collision}}$	−10

**Table 3:** Joint Range of Motion Chart.

Joint Number	Joint Name	Limit (rad)
0	L_LEG_HIP_YAW	−2.758~2.758
1	L_LEG_HIP_ROLL	−0.524~2.967
2	L_LEG_HIP_PITCH	−2.531~2.880
3	L_LEG_KNEE	−0.087~2.880
4	L_LEG_ANKLE_ROLL	−0.262~0.262
5	R_LEG_HIP_YAW	−2.758~2.758
6	R_LEG_HIP_ROLL	−2.967~0.524
7	R_LEG_HIP_PITCH	−2.531~2.880
8	R_LEG_KNEE	−0.087~2.880
9	R_LEG_ANKLE_ROLL	−0.262~0.262

**Table 4:** Hyperparameter configuration for PPO-based locomotion training.

Parameter	Value
Number of environments	4096
Learning rate	adaptive
Discount factor (γ)	0.996
GAE factor (λ)	0.95
Number of batches	4
gradient clipping	1
Clip range	0.2
Actor hidden layers	[512, 256, 128]
Critic hidden layers	[512, 256, 128]
SRM network	[512, 256, 128]
SPM network	[512, 256, 128]
$h_{1}$, $h_{2}$	59, 4

**Table 5:** Performance comparison of different methods in the indoor stair-climbing scenario. Each method was tested 50 times under identical conditions.

Method	Falls	Slips	Up-Failures	Success Rate (%)
LSWM	0	2	1	94.0
SRPM	2	5	2	82.0
SRM	1	7	4	76.0
DreamWaq	5	8	7	60.0
DWL	5	10	6	58.0

**Table 6:** Comparison of COT over 4 s for different algorithms.

Method	COT (J/kg/m)
DWL	0.89
SRM	0.85
DreamWaq	0.87
SRPM	0.82
LSWM	0.78

## Data Availability

The original contributions presented in this study are included in the article material. Further inquiries can be directed to the corresponding author.
